# Taste Compound Generation and Variation in Chinese Water Chestnut (*Eleocharis dulcis (Burm.f.) Trin. ex Hensch.*) Processed with Different Methods by UPLC-MS/MS and Electronic Tongue System

**DOI:** 10.3390/foods11233869

**Published:** 2022-11-30

**Authors:** Guanli Li, Hui Nie, Shuangquan Huang, Xiaochun Li, Shujie Wu, Xiaoxian Tang, Mubo Song, Yanghe Luo

**Affiliations:** 1 Guangxi Key Laboratory of Health Care Food Science and Technology, Institute of Food Science and Engineering Technology, Hezhou University, Hezhou 542899, China; 2 School of Food Science and Technology, Dalian Polytechnic University, Dalian 116034, China

**Keywords:** Chinese water chestnut, steaming and cooking process, UPLC-MS/MS, electronic tongue system, taste compounds, formation pathway

## Abstract

Chinese water chestnut (CWC) is popular among consumers due to its unique flavor and crisp and sweet taste. Thus far, the key substances affecting the taste compound of CWC are still unclear. In this study, we used UPLC-MS/MS and an electronic tongue system to study the effects of four typical steaming and cooking methods, cooking without peel for 10 min (PC), steaming without peel for 15 min (PS), cooking with peel for 30 min (WPC), steaming with peel for 30 min (WPS), on the taste compound generation and variation of CWC, and revealed the secret of its crisp and sweet taste. The results show that the electronic tongue can effectively identify the taste profile of CWC, and the effective tastes of CWC were umami, bitterness, saltiness, and sweetness. We screened 371 differential compounds from 640 metabolic species. Among them, nucleotides and their derivatives, carbohydrates, organic acids and their derivatives, and amino acids and their derivatives are closely related to the key taste of CWC, and these compounds affected the taste of CWC through six related metabolic pathways: oxidative phosphorylation and purine metabolism; alanine, aspartate, and glutamate; bile secretion; amino sugar and nucleotide sugar metabolism; the phenylpropane pathway; and toluene degradation. This study reveals the potential metabolic causes of taste compound generation and variation in the taste of CWC.

## 1. Introduction

*Eleocharis dulcis* (*Burm.f.*) Trin. ex Hensch. (Chinese water chestnut: CWC) is a characteristic economic crop originating in southern China that is now widely distributed around the world but is most famous in Guangxi [1,2]. CWC is mostly planted in paddy fields and harvested at the winter solstice when displaying a smooth surface, oblate bulb, purple-black skin, and white and delicate pulp. The CWC tastes crisp and delicious, with a unique flavor and rich nutrition, it has been known as the “Underground Snow Pear” since ancient times [3,4], and is believed to be rich in health-promoting compounds, such as flavonoids and polyphenols [5]. Therefore, CWC is a popular seasonal vegetable.

CWCs are rich in bioactive metabolites that influence their taste and aroma [6,7]. In addition, variety, processing time and temperature, and steaming and cooking methods are also important factors affecting CWC flavor quality. Zhan et al. believed that steaming with the peel could protect the quality of CWC and was a more appropriate processing method [8]. In our previous research, we found that a large number of volatile odor substances were produced during the CWC steaming and cooking process, giving them a special flavor [7]. In addition, important secondary metabolites created while steaming and/or cooking CWCs, such as phenylpropanoids and flavonoids, have attracted significant attention [6]. While there has been considerable interest in identifying the bioactive compounds and factors that contribute to CWC processing qualities, there appear to be no reports on the flavor characteristics and the effect of cooking on the constituent taste compounds of CWCs.

Taste is one of the most important quality attributes of food that affects consumer eating satisfaction [9]. Compounds that contribute to taste are nonvolatile and are generally derived from amino acids, nucleotides, organic acids, carbohydrates, etc. [10,11]. Furthermore, most of these compounds not only have nutritional functions that are beneficial to human health but are also important precursors of flavor substances [12]. At present, the analysis of food taste is mostly judged by sensory taste; this method is subjective and biased by personal preference [7]. The electronic tongue and high-performance liquid chromatography-mass spectrometry (UPLC-MS/MS) are the most powerful methods for studying food taste [13,14]. By simulating the human tongue, the electronic tongue can identify and detect complex taste substances and can replace sensory assessors to evaluate food taste. Electronic tongues are widely used in meat, fruit and vegetable, and seafood evaluation due to the fact that it is fast, simple, safe, etc. [15,16]. UPLC-MS/MS has the advantages of having a high resolution and high sensitivity, and allowing for the simultaneous quantitative analysis of hundreds of compounds, making it an ideal choice for metabolite identification [14,17,18]. In this study, we used UPLC-MS/MS and electronic tongue technology to systematically explore the effects of CWC steaming and cooking on metabolites and to elucidate the chemical basis for CWC taste quality, with the goal of providing novel insights into the taste and quality changes in CWC due to thermal processing.

## 2. Materials and Methods

### 2.1. Plant Material and Chemicals

*E. dulcis* (*Burm.f.*) Trin. ex Hensch. were fresh, free from rot, uniform in size, and purchased from a market in Hezhou, Guangxi, PR China.

Methanol, acetonitrile, and ethanol were HPLC grade and purchased from Merck (Darmstadt Germany). The HPLC standards were purchased from Sigma-Aldrich (St. Louis, MO, USA). The standard solution was stored at 4 °C.

### 2.2. Sample Preparation

Fresh CWC samples (FF) that were cooked without peel for 10 min (PC), steamed without peel for 15 min (PS), cooked with peel for 30 min (WPC), or steamed with peel for 30 min (WPS) were prepared according to reference [6]. The five CWC samples were stored at −80 °C until needed.

(1) Five CWC samples were freeze-dried in a freeze-dryer, and the dry samples were then ground for 1.5 min at 30 Hz to powder using a grinder (MM 400, Retsch, Germany).
(2) A 100.00 mg powder sample was accurately weighed and dissolved in 0.6 mL of 70% methanol.
(3) The dissolved sample was placed in a refrigerator at 4 ℃ overnight, during which time it was vortexed six times to improve the extraction rate.
(4) After centrifugation for 10 min at 10,000× *g*, the supernatant was extracted and filtered with a 0.22 μm pore size microporous membrane and stored in an injection bottle for UPLC-MS/MS analysis. Each experiment was repeated three times.

### 2.3. Ultra-Performance Liquid Chromatography-Tandem Mass Spectrometry (UPLC-MS/MS)

The data acquisition instrument system included devices for ultra-performance liquid chromatography (UPLC, Shim-pack UFLC SHIMADZU CBM30A, https://www.shimadzu.com.cn/ (accessed on 23 October 2022)) and tandem mass spectrometry (MS/MS, Applied Biosystems 4500 QTRAP, http://www.appliedbiosystems.com.cn/ (accessed on 23 October 2022)).

#### 2.3.1. Collection Conditions of UPLC-MS/MS

UPLC-MS/MS and qualitative and quantitative analyses were performed according to the reference [6].

The liquid phase conditions were the following:

(1) Chromatographic column: Waters ACQUITY UPLC HSS T3 C18 1.8 µm ×2.1 mm × 100 mm.
(2) Mobile phase: Phase A was ultrapure water (with 0.04% acetic acid), and phase B was acetonitrile (with 0.04% acetic acid).
(3) Elution gradient: The proportion of phase B was 5% at 0.00 min, and this linearly increased to 95% within 10.00 min and was maintained at 95% for 1 min; phase B was reduced to 5% at 11.00–11.10 min and balanced at 5% up until 14 min.
(4) The flow rate was 0.35 mL/min, the column temperature was 40 °C, and the injection volume was 4 µL.

The mass spectrometry conditions were the following: the electrospray ionization (ESI) temperature was 550 ℃, the mass spectrometer voltage was 5500 V, the curtain gas (CUR) was 30 psi, and the collision-activated dissociation (CAD) parameter was set to “high”.

#### 2.3.2. Qualitative and Quantitative Compound Analysis

Based on the self-built MWDB (Metware database), the compounds were qualitatively assessed according to the secondary spectrum information. Compound quantification was accomplished by the multiple reaction monitoring (MRM) analysis of triple-quadrupole mass spectrometry.

### 2.4. Electronic Tongue Analysis

The electronic tongue system (INSENT, TS-5000Z, Japan) comprised six potentiometric chemical sensors (CA0, C00, AE1, GL1, AAE, and CT0) and was chosen to analyze the flavor of the five sample groups. The electronic tongue uses an artificial lipid membrane sensor with wide-field selection specificity to mimic the taste sensations experienced by living organisms by detecting changes in the membrane potential generated by the electrostatic or hydrophobic interactions between various taste substances and the artificial lipid membrane. The six potentiometric chemical sensors showed different sensitivities to the five basic tastes (umami, sourness, saltiness, bitterness, and sweetness) in the samples. The characteristics and performance of each sensor are shown in Table 1.

### 2.5. Statistical Analysis

The UPLC-MS/MS and electronic tongue data were analyzed using Excel 2016 and IBM SPSS Statistics 25. Principal component analysis (PCA), Orthogonal partial least squares discriminant analysis (OPLS-DA), and Cluster analysis (HCA) were performed using R software (http://www.r-project.org/ (accessed on 23 October 2022)), and the pictures were drawn using Origin Pro and Adobe Illustrator CC. The chemical analyses were repeated at least three times.

## 3. Results and Discussion

### 3.1. The Results of the Electronic Tongue

#### 3.1.1. Identification of Effective Taste Indicators

**Figure 1:**
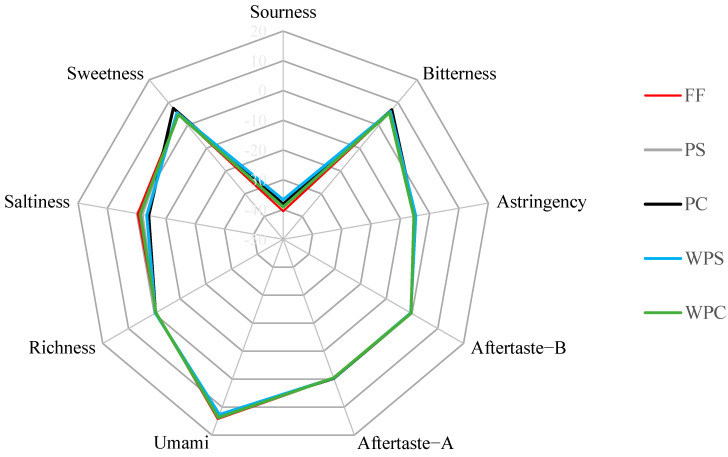
Spider plot for the electronic tongue sensory score based on the taste sensing system in CWC of different steaming and cooking process.

The electronic taste sensor simulates the human taste perception mechanism and quickly and objectively judges the five basic tastes (umami, sourness, saltiness, bitterness, and sweetness). This assessment can be used for quality control and sample monitoring in the food industry [15,16]. In this study, according to refsol, the tasteless point of each taste is determined. The reference solution consisted of KCl and tartaric acid, so the tasteless point of sourness was −13 and the tasteless point of saltiness was −6. The tasteless point of all other indicators was 0, except for sourness and saltiness, and values that were larger than these tasteless points were considered meaningful [9]. The outputs of the 9 taste sensors are shown in Table 2. Based on these data, a spider plot was made to illustrate the evolution of different taste components during CWC processing (Figure 1). As shown in Figure 1, the electronic tongue taste sensor was able to respond to the taste of each CWC sample processed with the various chosen methods, but its sensitivity was different for each sample. Among them, the sensor response value of the freshness was the largest, followed by sweetness, bitterness, saltiness, and the other taste components, including sourness, astringency, aftertaste-B, aftertaste-A, and richness, which had values less than their tasteless points, indicating that the effective taste indices of CWC were umami, bitterness, saltiness, and sweetness.

**Figure 2:**
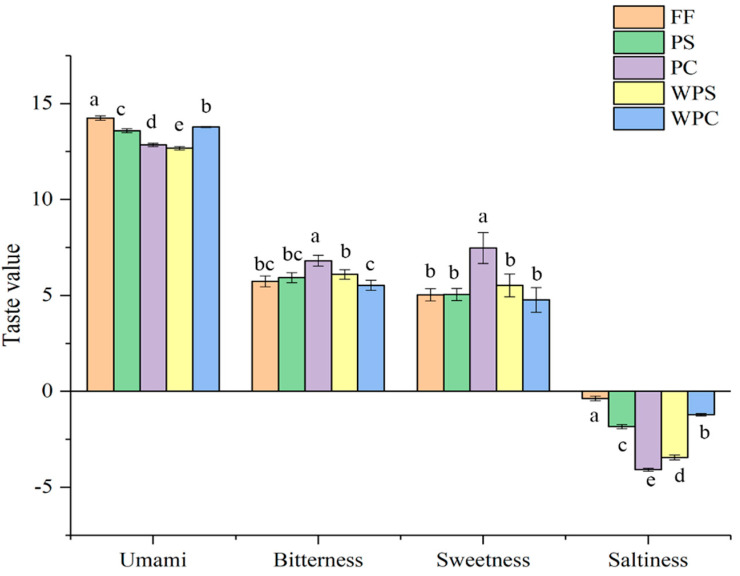
Bar chart of the main taste indicators for the electronic tongue. Bars with different letters within the same bar differ significantly (*p* < 0.05).

There were significant differences in the effective taste indices (bitterness, saltiness, umami, and sweetness) for different CWC processing methods (Figure 2). Compared with FF, the umami and saltiness values of the other four CWC groups decreased significantly, while the sweetness and bitterness values increased significantly. At the same time, we can see that the dominant taste of FF, WPC, and PS samples was umami, and the bitterness, sweetness, and saltiness of the WPS and PC samples were more prominent. However, we did not taste obvious bitterness and saltiness when eating CWC, which might be because the umami value was significantly positively correlated with the saltiness value (*p <* 0.01), and there was also a significant positive correlation between sweetness and bitterness (*p <* 0.01) (Table 3), indicating a synergistic effect between bitterness and sweetness, and saltiness and umami, thus giving CWC a flavor profile characterized by umami and sweetness. The characteristic taste of CWC is not the result of a single class of substances but the result of the joint action of amino acids and their salts, taste nucleotides and their salts, and organic acids and peptides.

#### 3.1.2. PCA Analysis

**Figure 3:**
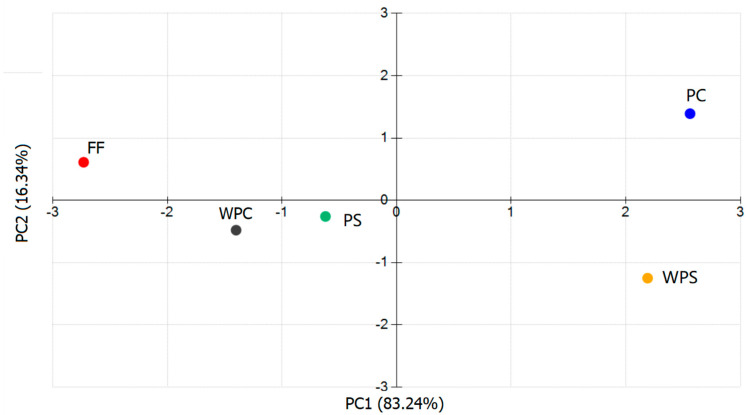
The scores plot of PCA for the electronic tongue.

PCA is a classical statistical analysis method in an unsupervised mode that performs principal component analysis on the main taste indices detected by the electronic tongue [19]. The results are shown in Figure 3. The PCA score plots indicated the relationship between variables using the variable information based on PC1 and PC2. The results showed that the cumulative contribution rate of the first principal component (PC1 83.24%) and the second principal component (PC2 6.34%) was 89.58%, which is greater than 80.0%, indicating that the electronic tongue PCA could reflect the overall information of the sample taste. Figure 3 shows that the fresh samples are mainly distributed in the first quadrant, which is clearly different from the CWC samples obtained using processing methods, indicating that CWC produces special taste substances after cooking. WPC and PS are distributed in the second quadrant and are close to each other, and the results are completely distinguished with no overlapping area between them. The results for WPC and PS are far from PC and WPS, indicating that there are significant differences in the taste characteristics of the CWC samples produced using different processing methods.

In conclusion, the electronic tongue combined with the recognition mode can effectively distinguish CWC samples produced using different processing methods, but the key substances affecting their characteristic taste still need to be further analyzed in combination with UPLC-MS/MS.

### 3.2. UPLC-MS/MS Metabolomics Analysis

To better understand the taste differences in the CWC samples produced using different steaming and cooking processes, we performed widely targeted UPLC-MS/MS-based on the metabolite profiling of five CWC samples, which were produced using different steaming and cooking processes (FF, PC, PS, WPC, and WPS). In total, 640 metabolites were identified, including a large number of metabolites likely to contribute to taste (97 organic acids and their derivatives, 84 amino acids and their derivatives, 48 nucleotides and their derivatives, and 17 carbohydrates) as well as other primary and secondary metabolites (Supplementary Table S1).

#### 3.2.1. Key Nonvolatile Taste Components Obtained Using Different CWC Processing Methods

**Figure 4:**
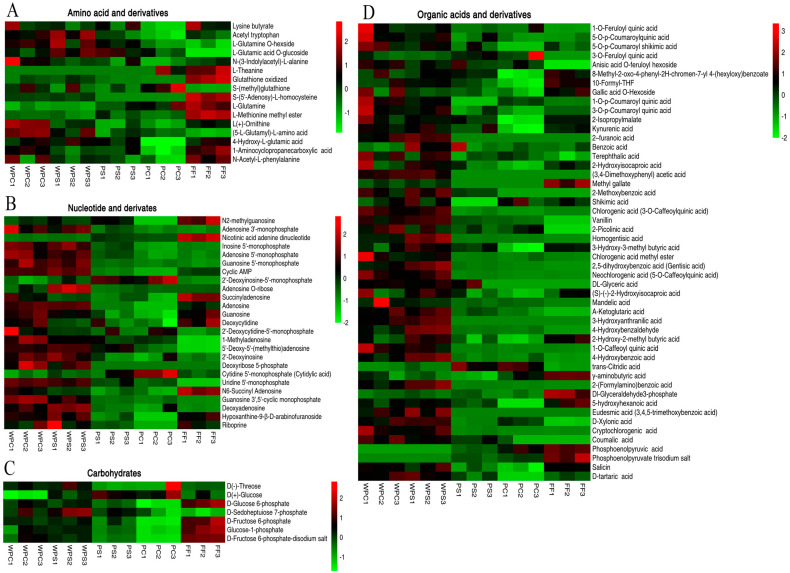
Heat map visualization of non-volatile differential metabolites in five groups of CWC samples. Each column represents a sample. The abscissa represents different experimental groups. The ordinate represents the different metabolites compared by group; from left to right, boiled sample WPC, WPS, PS, PC, FF. The color indicates the relative expression of metabolites, from low (green) to high (red). (**A**) Amino acids and derivatives, (**B**) Nucleotides and derivatives, (**C**) Carbohydrates, (**D**) Organic acids and derivatives.

To clarify the influence of different processing methods on nonvolatile taste compounds in CWC, we used variable importance projection VIP ≥1, combined with univariate analysis difference fold change ≥2 or fold change ≤0.5 to screen key flavor substances [12]. A total of 371 differential compounds were screened from 640 compounds obtained from five CWC samples (Supplementary Table S2). We focused on classes of metabolites likely to be major contributors to taste. Figure 4 shows heatmaps presenting the main components that affect key tastes, and the color of the heatmap represents the relative metabolite content. High and low metabolite expression levels are presented in red and green, respectively. The color change from green to black to red represents a gradual increase in the relative metabolite content [10,15].

There were significant differences in the content and species of compounds obtained using different steaming and cooking processes (Figure 4A–D). The organic acid, organic acid derivative, and carbohydrate content in FF were relatively high, while those in the other samples were relatively low. This may be due to the Maillard reaction between these two substances during high-temperature steaming and cooking, which produces new flavor substances [20], thus leading to the flavor differences between CWC samples obtained using different steaming and cooking processes. Organic acids and their derivatives and nucleotides and their derivatives in WPS and WPC displayed more red, while PS and PC showed almost no red. This may be due to the transfer of compounds in the CWC peel to the pulp during the steaming and cooking process, leading to a difference in the flavor of the CWC samples when using different steaming and cooking methods [6]. This result indicated that the characteristics and taste of CWC in different processing methods were closely related to these amino acids and their derivatives, organic acids and their derivatives, and nucleotides and their derivatives.

#### 3.2.2. Amino Acids and Their Derivatives

Amino acids and their derivatives are important components of living organisms and important precursors for the formation of taste substances [12]. The taste characteristics of amino acids can be divided into four main categories: umami, bitter, sweet, and tasteless [15]. Hydrophilic amino acids are primarily associated with umami and sweet tastes, whereas hydrophobic amino acids are primarily associated with a bitter taste [13]. As evinced by the cluster analysis (Figure 4A), we clearly and intuitively showed changes in 16 different amino acids across the different CWC sample processing methods. Among them, l-glutamine is a typical umami amino acid, which is also found to be the main flavor substance in mushrooms [12]. Interestingly, the l-glutamine content decreased in the order of PC, PS, WPC, and WPS, while the l-(+)-ornithine content increased in the order of PC, PS, WPS, and WPC, indicating that there may be a synergistic effect between the two, which together endows CWCs with an umami and sweet taste [12]. L-theanine, as a derivative of glutamine, has umami and caramel flavors and can synergistically improve the umami taste of sodium glutamate [21]. Compared with FF, the L-theanine content decreased significantly in WPC, WPS, and PS samples, but not in PC, which may be due to the reaction of L-theanine with glucose to produce deoxy-l-mandoline-l-pyrrolulose during processing, giving CWCs a sweet taste. The accumulation of these amino acids in CWCs may be attributable to proteolysis products [22].

#### 3.2.3. Nucleosides and Their Derivatives

Nucleotides and their related compounds are closely associated with the taste of CWCs and are mainly derived from the metabolism of adenosine triphosphate (ATP). ATP is degraded to adenosine diphosphate (ADP) and reduced to adenosine monophosphate (AMP), which is then followed by a reduction to disodium inosine-5-monophosphate (IMP) [22]. Across the five CWC sample groups, we found 25 nucleotides and nucleotide derivatives with significant differences (Figure 4B). Among them, adenosine 5′-monophosphate (AMP), guanosine 5-monophosphate (GMP), inosine 5′-monophosphate (IMP), and uridine 5′-monophosphate (5-UMP) have the most typical umami taste. AMP not only has a sweet taste, fresh taste, and other qualities but can also inhibit the bitterness of aquatic products and is a good flavor enhancer [12]. IMP has been proven to be one of the main taste compounds that cause the delicious taste of globefish. The addition of IMP to sweet amino acids also has the effect of enhancing sweetness [22]. GMP is considered an important flavor substance of dried mushrooms [23]. At the same time, these taste nucleotides can produce synergistic effects with ASP, Glu, etc., to produce a stronger flavor effect. Compared with FF, the nucleotide contents in the CWCs after thermal processing showed an overall upwards trend with certain differences between the different processing methods. The contents of IMP, AMP, and GMP in the WPC and WPS samples increased significantly, but there was no difference in PC and PS samples. Overall, thermal processing increased the IMP, AMP, and GMP contents and enhanced the umami taste of CWCs.

NAD^+^ and adenosine and their derivatives are downstream products of nucleotide degradation, of which adenosine is not only an intermediate for the synthesis of ATP, AMP, and other substances, but also involved in a variety of important metabolism and functional regulation processes beneficial to human health [12]. Compared with FF, the NAD^+^ content decreased significantly in the four groups of processed samples, while the adenosine content decreased significantly in PC, and the adenosine content did not change significantly in the other three groups of processed samples. The change in the content of these metabolites showed that the macromolecular nucleotides in CWC were degraded after thermal processing and may change the taste of CWC.

#### 3.2.4. Carbohydrates

The carbohydrate content is an important index for evaluating the quality of fruits and vegetables and is closely related to the taste and smell of fruits and vegetables [10]. A total of 17 kinds of sugars were identified from the five CWC sample groups, of which 7 had significant biological differences (Figure 4C). Compared with FF, these 7 carbohydrate compounds generally showed a downward trend, which may be due to the Maillard reaction between sugars and amino acids during high-temperature steaming and cooking, so sugars are not only flavor substances but also precursors of volatile flavor substances [20]. Interestingly, the sweet-related compounds in CWC include glucose and d-(-)-threose, and the sugars related to sweetness are mainly glucose and d-(-)-threose. The content of glucose in WPC decreased significantly, but the content of glucose in other samples did not change much. The content of d-(-)-threose was downregulated in PS samples, but there was no significant difference in other samples. These results suggested that they may be the key compounds that distinguish the taste of CWC prepared using different processing methods.

#### 3.2.5. Organic Acids and Their Derivatives

Organic acids play an important role in the taste quality of fruits and vegetables [24]. We identified 97 kinds of organic acids from the five CWC sample groups, of which 50 had significant biological differences (Figure 4D). Among them, d-xylonic acid has a refreshing and sweet taste, and its content in the WPC, WPS, and PC samples increased significantly, while its content in the PS samples did not change much, suggesting that d-xylonic acid may be the characteristic flavor substance of CWCs. Interestingly, shikimic acid, d-(-)-tartaric acid, and benzoic acid all have a strong sour taste, but the electronic tongue analysis showed that a sour taste was not a distinctive taste of CWCs, so it may be the coordination of umami and sweet tastes that determine the unique CWC flavor.

In addition, we also detected other organic acid compounds with high content and strong flavor, such as vanillin, which was the compound with the highest content shared by the four steamed CWC sample groups. Vanillin has a strong milk flavor and has an important contribution to the flavor composition [25]. A higher 4-hydroxybenzaldehyde content has an aromatic smell and contributes to the flavor of CWC. Cryptochlorogenic acid was detected only in WPS and WPC and is a glycosidic aroma precursor, and its isomer chlorogenic acid has a fragrance; furthermore, cryptochlorogenic acid can also be oxidized to give pyrrole, pyrazine, and other volatile aroma components [21].

### 3.3. KEGG Classification and Enrichment Analysis of Differential Metabolites

**Figure 5:**
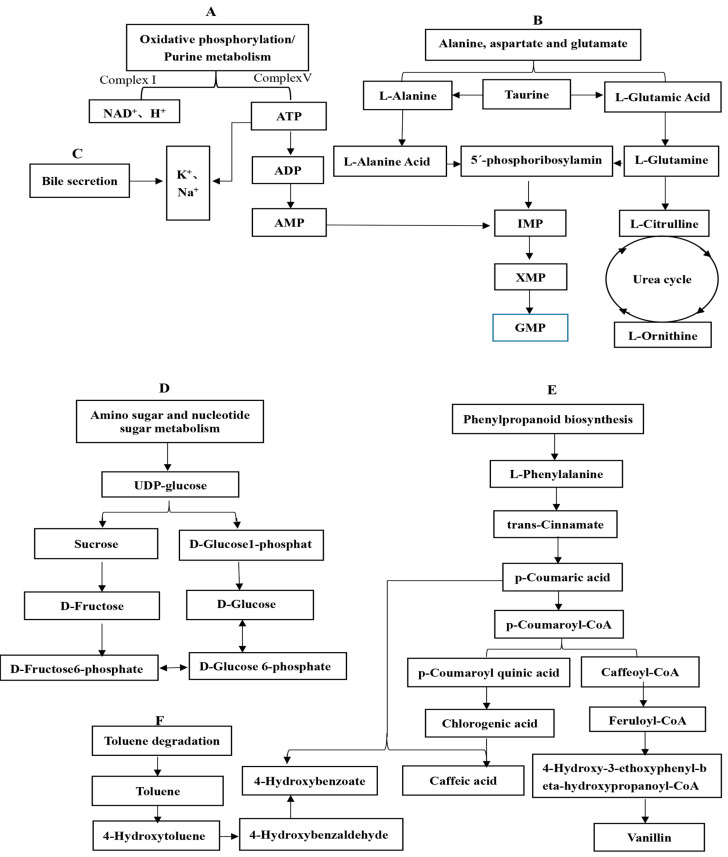
The formation pathway of CWC key taste substances under different steaming and cooking processes. (**A**) Oxidative phosphorylation and Purine metabolism; (**B**) Alanine, aspartate, and glutamate; (**C**) Bile secretion; (**D**) Amino sugar and nucleotide sugar metabolism; (**E**) Phenylpropane pathway; (**F**) Toluene degradation.

Metabolic pathway analysis is a useful method for directly analyzing the internal connections of metabolites and can reconstruct the biochemical reaction network [26,27]. The main pathways of CWC metabolism are summarized and outlined in Figure 5. The effective taste indices of CWC were umami, bitterness, saltiness, and sweetness, which affect the taste of CWC through six related metabolic pathways. These five pathways include the following: oxidative phosphorylation and purine metabolism (A); alanine, aspartate, and glutamate (B); bile secretion (C); amino sugar and nucleotide sugar metabolism (D); the phenylpropane pathway (E); and toluene degradation (F).

In A, complex I and complex V generate NAD^+^ and ATP, respectively, under the catalysis of NADH dehydrogenase and ATP synthase, in which the reduction of ATP to ADP and the subsequent reduction to AMP and then IMP regulate the taste of CWC. At the same time, ATP can also participate in the C pathway to produce K^+^ and Na^+^, so the electronic tongue detects a subtle salty taste in CWC. In addition, a fresh taste can also regulate the taste of CWC by transforming taurine into 2-ketoglutarate and then producing glutamate and glutamine under the action of glutamate synthase and glutamine synthetase, respectively. In B, l-glutamine, which has a delicious taste, generates l-citrulline under the action of a series of enzymes and enters the urea cycle, regulating the l-(+)-ornithine content and then regulating the sweetness of CWC.

In D, the intermediate UDP glucose can regulate the generation of d-glucose and sucrose. Among them, the d-glucose content in WPC decreases significantly, indicating that the metabolic pathway of WPC is transformed to glucose 6-phosphate at this time. However, d-glucose and d-fructose 6-phosphate can be mutually transformed, so the sweetness of WPC samples is basically unchanged. The d-fructose 6-phosphate and d-fructose 6-phosphate contents in PC samples decreased, and they could also be transformed into each other, so the sweetness of PC samples changed little.

In E, trans-cinnamic acid, an intermediate, can regulate the formation of vanillin and chlorogenic acid, the flavor substances of CWC. In WPS and WPC, the vanillin and chlorogenic acid contents increased with an increasing trans-cinnamic acid content, thus giving WPS and WPC a rich fragrance. P-coumaric acid, an intermediate of E, participates in F by regulating the formation of 4-hydroxybenzoic acid. After a series of dehydrogenation and catalytic reactions, F produces the main flavor substance of CWC: 4-hydroxybenzaldehyde.

### 3.4. Discussion

CWC is famous for its unique flavor and crisp and sweet taste [7]. In the early stage of this study, we used an electronic tongue and SPME-GC-MS to study the volatile substances created using four typical CWC cooking and processing techniques, namely, peeling then boiling for 20 min (PC), peeling then steaming for 20 min (PS), boiling with peel for 30 min (WPC), and steaming with peel for 30 min (WPS), and we identified the characteristic flavor substances of CWC [6,7]. However, the characteristic taste components created after using different cooking and processing CWC are still unclear.

In this study, we analyzed the effects of amino acids and their derivatives, nucleotides and their derivatives, organic acids and their derivatives, carbohydrates, and other important differential compounds on the characteristic taste of CWC. However, alkaloids, flavonoids, terpenoids, bitter amino acids, and other differential compounds also have certain effects on the characteristic taste of CWC. The research shows that flavonoid glycosides have an astringent taste that causes the dry and soft convergence of the mouth, and their taste threshold is very low, so their content changes contribute to the formation of the special taste of CWC [21]. High contents of naringin, quercetin, and catechins in WPC and WPS result in an obvious bitter taste. Naringin is a sweetener when it is ring-opened and hydrogenated to form dihydrochalcones under alkaline conditions, and its sweetness can reach 2000 times that of sucrose. Naringin and catechins are easily oxidized to form colored substances during processing, which may be the key substances leading to the yellowing of CWC [9,28]. At the same time, catechins are prone to stereoisomerism, reducing the bitter taste and further increasing the sweet taste of CWC. Aesculin is a bitter agent that is hydrogenated and converted into dihydrochalcone during heating. Its sweetness is 150 times that of sucrose, giving WPC and WPS a sweet taste. Therefore, aesculin may participate in the formation of the taste of steamed and boiled samples with peel. In addition, when amino acids are unbalanced in hydrolysis or casein is hydrolyzed, some bitter amino acids will be produced, such as proline, leucine, tyrosine, l-methionine, l-tryptophan, or l-(+)-arginine [12], but no bitter taste was found in the sensory evaluation. However, the electronic tongue was able to recognize the bitter taste of CWC, which may be because electronic tongues are more sensitive than human tongues [29].

A salty taste is also one of the effective taste indicators of CWC and is mainly caused by inorganic ions [30]. Through the analysis of the KEGG pathway diagram, it was found that ATP can produce K^+^ and Na^+^, giving CWC a slightly salty taste [28]. However, inorganic ions were not detected in this study, so the important compounds causing the saltiness of CWC are not clear, and further research is needed.

In summary, amino acids, organic acids, nucleotides, and carbohydrates interacted with each other with a synergistic or suppressive effect and contributed to the overall taste and flavor of CWC.

## 4. Conclusions

In this study, we clarified the processes of taste compound generation and variation in CWC after using different cooking processes using UPLC-MS/MS and an electronic tongue, with the goal of revealing the secret of its taste. The results show that the electronic tongue can effectively distinguish CWC samples prepared using different processing methods, and the effective taste indices of CWC were umami, bitter, salty, and sweet. The difference in the composition and concentrations of organic acids and their derivatives, amino acids and their derivatives, nucleosides and their derivatives, and carbohydrates might be the underlying causes of the differences in taste among the CWC produced using different steaming and cooking processes, and these compounds affected the taste of CWC through six related metabolic pathways, including oxidative phosphorylation and purine metabolism; alanine, aspartate, and glutamate; bile secretion; amino sugar and nucleotide sugar metabolism; the phenylpropane pathway; and toluene degradation. This work provides new insights into the compositions and taste differences of metabolites in CWC.

## Figures and Tables

**Table 1:** Features and performance of TS-5000Z electronic tongue sensor.

Sensor Name	Evaluable Taste	
	Basic Flavor (Relative Value)	Aftertaste (CPA Value)
Freshness sensor (AAE)	Fresh taste caused by amino acids and nucleic acids	Freshness richness (Sustainable perceived freshness)
Saltiness sensor (CT0)	Salty taste caused by inorganic salts such as table salt	NA
Sour sensor (CA0)	Sour taste caused by acetic acid, citric acid, tartaric acid, etc.	NA
Bitterness sensor (C00)	The taste caused by bitter substances is perceived as richness at low concentrations	Bitter aftertaste (bitter taste of beer, coffee, and other general foods)
Astringency sensor (AE1)	The taste caused by astringent substances is perceived as an irritating aftertaste at low concentration	Astringent aftertaste (astringency of tea, red wine, etc.)
Sweetness sensor (GL1)	Sweetness caused by sugar or sugar alcohol	NA

**Table 2:** Taste response intensity in CWC of different steaming and cooking process.

CH	Sourness	Bitterness	Astringency	Aftertaste-B	Aftertaste-A	Umami	Richness	Saltiness	Sweetness
Tasteless	−13	0	0	0	0	0	0	−6	0
FF	−40.56	5.73	−5.43	−0.3	−0.32	14.25	−0.66	−0.37	5.03
PS	−38.96	5.93	−5.19	−0.45	−0.37	13.59	−0.54	−1.84	5.05
PC	−38.2	6.81	−5.09	−0.43	−0.26	12.85	−0.45	−4.09	7.47
WPS	−36.71	6.09	−4.79	−0.48	−0.35	12.67	−0.4	−3.44	5.52
WPC	−39.15	5.52	−5.42	−0.38	−0.43	13.78	−0.65	−1.22	4.76

**Table 3:** Correlation analysis of CWC effective taste index in different steaming and cooking processes.

	Bitterness	Umami	Saltiness	Sweetness
Bitterness	1	−0.723	−0.865	0.972 **
Umami	−0.723	1	0.968 **	−0.647
Saltiness	−0.865	0.968 **	1	−0.810
Sweetness	0.972 **	−0.647	−0.810	1

Note: ** Correlation is significant at the 0.01 level (2-tailed).

## Data Availability

The data are available from the corresponding author.

## Supplementary Materials

The following supporting information can be downloaded at: https://www.mdpi.com/article/10.3390/foods11233869/s1: Supplementary Table S1. 640 compounds in 5 groups of CWC samples. Supplementary Table S2. Difference compounds in five groups of CWC samples.
